# Solving Inverse Electrocardiographic Mapping Using Machine Learning and Deep Learning Frameworks

**DOI:** 10.3390/s22062331

**Published:** 2022-03-17

**Authors:** Ke-Wei Chen, Laura Bear, Che-Wei Lin

**Affiliations:** 1 Department of BioMedical Engineering, National Cheng Kung University, Tainan City 70101, Taiwan; p86061354@ncku.edu.tw; 2 Electrophysiology and Heart Modelling Institute (IHU-LIRYC), Fondation Bordeaux Université, 33000 Bordeaux, France; laura.bear@ihu-liryc.fr; 3 Centre de Recherche Cardio-Thoracique de Bordeaux, INSERM U1045, Université de Bordeaux, 33600 Pessac, France

**Keywords:** electrocardiographic imaging (ECGi), deep learning, machine learning, inverse problem, Fully Connected Neural network (FCN), Long Short-term Memory (LSTM), Convolutional Neural Network (CNN)

## Abstract

Electrocardiographic imaging (ECGi) reconstructs electrograms at the heart’s surface using the potentials recorded at the body’s surface. This is called the inverse problem of electrocardiography. This study aimed to improve on the current solution methods using machine learning and deep learning frameworks. Electrocardiograms were simultaneously recorded from pigs’ ventricles and their body surfaces. The Fully Connected Neural network (FCN), Long Short-term Memory (LSTM), Convolutional Neural Network (CNN) methods were used for constructing the model. A method is developed to align the data across different pigs. We evaluated the method using leave-one-out cross-validation. For the best result, the overall median of the correlation coefficient of the predicted ECG wave was 0.74. This study demonstrated that a neural network can be used to solve the inverse problem of ECGi with relatively small datasets, with an accuracy compatible with current standard methods.

## 1. Introduction

### 1.1. Inverse Electrocardiographic Mapping

Studying the electrical activity of the heart is important for clinicians to make diagnoses or for monitoring. By placing electrodes on the body’s surface, such as the chest and limbs, we can reveal the electrical activity remotely and non-invasively. The graph data we gather are referred to as electrocardiography (ECG or EKG) data. Recordings can also be invasive, gathering potential information directly from the endocardium through a catheter. The information gathered this way is referred to as an intracardiac electrogram. By combining the electrogram with its geometrical location, we can map the results to the endocardial surface, which is referred to as electroanatomical mapping.

Monitoring the heart’s electrical activity via body surface recordings is an indirect measurement method. For example, the typical 12-lead ECG system only shows 12 time series data points with the rough direction for each potential recording; therefore, it is difficult to locate the anatomical location of abnormalities. It will be very helpful if we could directly see the heart’s potential and its geometrical location within the heart. There have been many attempts to map body surface recordings to the heart; this process is called inverse electrocardiographic mapping. Usually, the target of mapping is the potential over the heart’s surface (epicardial potential). This mapping can also be referred to as electrocardiographic imaging (ECGi) [1].

### 1.2. The Importance of Inverse Electrocardiographic Mapping

For ablation of the arrhythmia source, it is important to identify the location of the abnormal rhythm. Traditionally, this is achieved by electroanatomical mapping, which is carried out by recording the potential information of the endocardium through a catheter. Currently, physicians need to perform this mapping directly by repeatedly touching the endocardium with a catheter, which can be time-consuming. Inverse electrocardiographic mapping can reduce the time and provide opportunities for pre-operation evaluations. This has already been used in the clinical field for pre-operation evaluations or quick analyses during operations. One example is the ECGi system (CardioInsight, Medtronic Inc., Minneapolis, MN, USA) [2].

### 1.3. Traditional Methods

Traditional methods for solving the problem generally involve two steps [3]. The first is called forward problem formulation [4,5], which uses the heart as an electromagnetic source. A cardiac source model can be constructed using Maxwell’s equations and geometrical information. It is usually put in a matrix A as follows:

(1) $$\emptyset_{T}=A\emptyset_{H}$$

where $\emptyset_{T}$ is the potential over the body’s surface in vector form, $\emptyset_{H}$ is the potential over the heart’s surface, and A is the matrix that transforms the heart’s surface potential into the body’s surface potential, which usually requires the geometrical information of the body and heart. The modeling of Matrix A is usually referred to as a forward problem.

The second step is to perform an inversion of matrix A as follows:

(2) $$\emptyset_{H}=A^{-1}\emptyset_{T}$$

where $A^{-1}$ is the inverse of matrix A. This problem is usually referred to as an inverse problem.

Multiple methods have been developed on the basis of this framework. The source of electrical activity can be directly modeled as the epicardial potential; it can also be modeled from the activation time. The first method is called the potential-based model and the second is the activation-based model. The Boundary Element Method (BEM) can be used to solve Matrix A, but it requires a mesh of the 3D geometry, which can sometimes be time-consuming and can introduce mesh-related defects. The meshless method of fundamental solutions (mMFS) was developed to solve this problem [6]. We can simply model the medium between the heart and body surfaces as a medium with consistent conductivity, which is referred to as homogeneous modeling. We can also consider the different conductivity within different tissue, such as lung, muscle, and fat; this model is referred to as an inhomogeneous model [7,8].

### 1.4. Problems Faced by the Current Methods

Despite the simplicity of this modeling method, it has many problems. For one, the inversion of Matrix *A* is not unique. Moreover, the inverse problem is ill-posed, which means that the prediction is subject to noise in the body’s surface potential; thus it requires further regularization [9].

The abovementioned problems may explain why the currently reported accuracy of the reconstructed potential is still not ideal, see Section 4.3. There are different ways to obtain this validation [10].

For the torso tank experiment, which used a tank as the body surface and a dog heart as the potential source, the median correlation coefficients could be up to 0.8 (the median correlation coefficient for the electrogram).

In an in situ animal study, the potential of the heart and body surfaces were simultaneously recorded in animals such as dogs or pigs. The current accuracy for such studies is around 0.7 (the median correlation coefficient of the activation time map) [11].

For validation in a clinical setting, the current result was also around 0.7 (the median correlation coefficient of the activation time map) for paced rhythm. The results were even worse for a normal QRS, which was 0.03 (the median correlation coefficient of the activation time map) [12].

### 1.5. Neural Network for Prediction

In recent years, neural networks have proven to be a useful tool for modeling data with complex relationships. Currently, only a few attempts have been made to solve the inverse problem with a neural network. In one study [13], data were collected from a torso tank setting using Time Delayed Neural Nets and Feed Forward Neural Nets (FFNNs). Their results were not ideal, with most having a median correlation coefficient of <0.5. There are two main problems, the first of which is overfitting. The model converges well in the training round but performs poorly in the testing round. The second problem is how to apply it to different subjects with a different heart and body geometry. This problem exists because the training data do not contain information on the geometry [13].

In our study, we built our model directly from data collected from an in situ animal study. To build a model that can be applied to subjects with different geometries, we created a data registration method to incorporate the geometric information into the data sequence.

## 2. Materials and Methods

### 2.1. Data Collection

Previous literature has illustrated the details of the data collection process [14]. A subset of the experimental data can be accessed online through the EDGAR project [15]. A short summary of the experiment is provided below. All surgical procedures were approved by the Animal Ethics Committee of the University of Auckland and conformed to the Guide for the Care and Use of Laboratory Animals (National Institutes of Health publication No. 85–23).

Two sets of data were collected. One included electrograms recorded from a vest wrapped around a pig. The electrograms record the potential change in the pig’s body surface. Another set included electrograms recorded from a sock wrapped around the heart. The electrograms showed the potential changes in the heart’s surface.

To place the recording leads, five anesthetized pigs (around 30–49 kg) underwent a midline sternotomy. A sock containing 239 unipolar silver wire electrodes was then placed around the heart. After the procedure, the chest was closed, and air was expelled from the lungs. Flexible strips containing electrodes were placed inside a vest that was wrapped around the pig’s torso. The epicardial and body surface potentials were then recorded simultaneously. The electrocardiograms were then recorded at a sampling rate of 2 kHz and a bandwidth limitation of 0.05–1000 Hz. The electrograms were further smoothed by moving the mean and synchronized average. To reduce the effect of potential shifting, we synchronously shifted all electrograms so that the mean of all potential values across all nodes at the beginning of the cycle equaled zero.

There were three pacing types: sinus rhythm, epicardial pacing, and endocardial pacing. The pacing sites were located all across the heart. Overall, we obtained 76 recordings, each of which contained around 10–20 beats. Table 1 shows the composition of the data. About 10–20% of the leads on the vest took poor recordings. We used linear interpolation to fill in the missing data.

### 2.2. Final Data Used in the Study

The data were further smoothed, first by the synchronized average, followed by the moving mean. For the synchronized average, each beat was extracted from the recordings at the same pacing site. The number of waves used for averaging ranged from around 5 to 10. We reviewed all the wave forms and removed the ones with poor quality. For the moving mean, we chose the last 20 data points for averaging. After this process, each pacing site or sinus rhythm contained only one electrocardiogram cycle (including QRS and T waves). The number of leads over the torso’s surface was around 150–170 and the total number of epicardial leads was 239. Table 1 shows the number of recordings from different pacing sites and recording leads. Note that the whole electrogram cycle was used for model training and testing. Unlike most studies, only the potential during ventricular activation was used.

**Figure 1:**
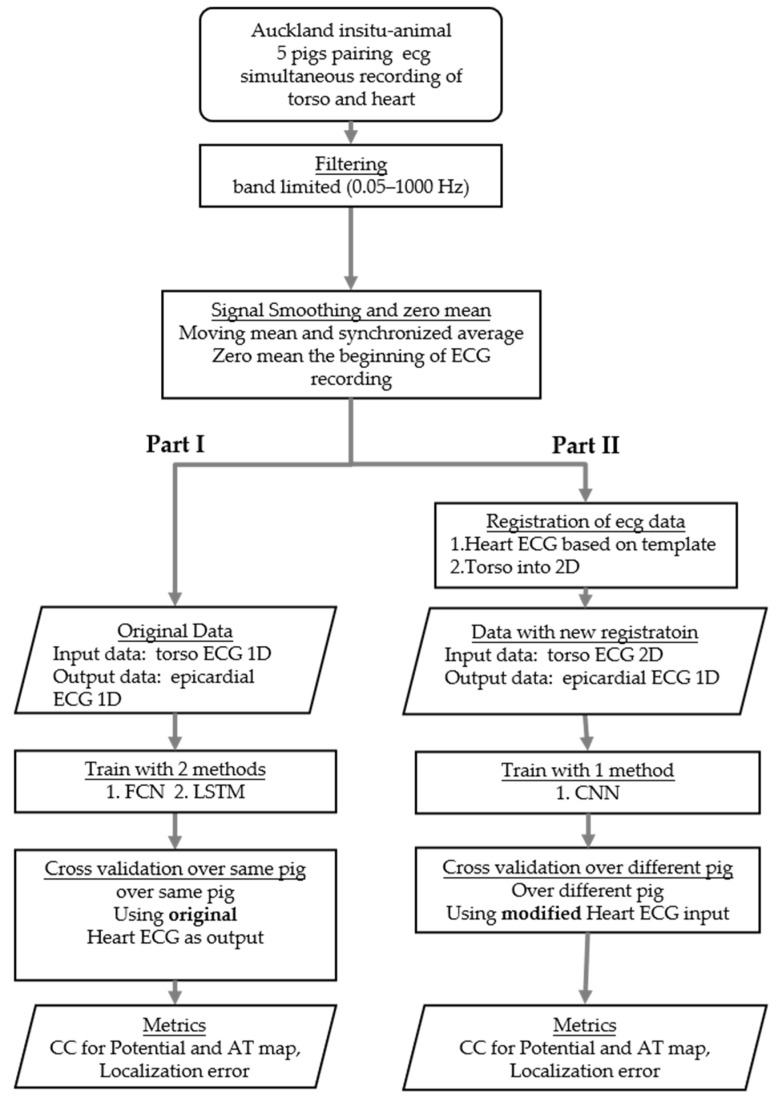
The overall study process. Part I indicates the study that trained the data for one pig only. Part II used data from all five pigs and had an additional step (registration of the electrogram data) that transformed the original data into a uniform data format. In terms of the metrics, the models were evaluated in two ways: (1) the correlation coefficients of the reconstructed electrogram and the recorded one, (2) the correlation coefficients for the AT map derived from the reconstructed and recorded electrograms. CC, correlation coefficients; AT, map activation time across all epicardial nodes.

Since each pig was a different experimental setting, there was no consistency regarding the number of recording leads over the torso, and different leads were placed at different geometrical locations. We could have trained the model directly from the data, but the model may only apply to data with the same experimental setting. Thus, we divided the experiment into two parts. In the first part, we only considered the problem within each individual pig, and we built the model for only one pig. In the second part, we incorporated a registration method to unify the data from different pigs. Figure 1 shows the overall study process.

### 2.3. Part I: Not Considering the Geometry

#### Model Selection

**Figure 2:**
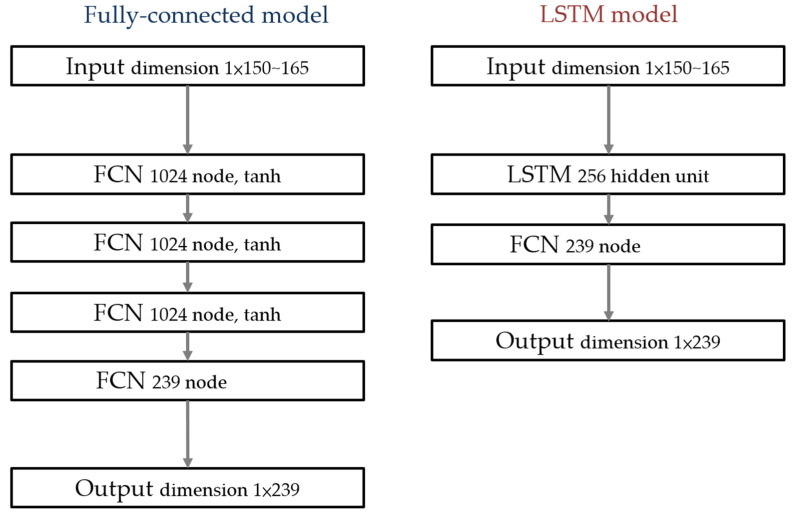
Models used in cross-validation over data from the same pigs as in Part II. (**Left**): The fully connected model used in this study; (**right**): the LSTM model used in this study. The input size ranged from 1 × 150–165 depending on the ECG recording vest used for the different pigs.

Two methods were used to establish the model. One was a neural network with a few fully connected layers using Hyperbolic Tangent as an activation function, as shown in Figure 1. The other was the Long Short-term Memory (LSTM) model. The structures of the models are shown in Figure 2.

### 2.4. Part II: Adding Geometrical Information

#### 2.4.1. Torso Node Registration

**Figure 3:**
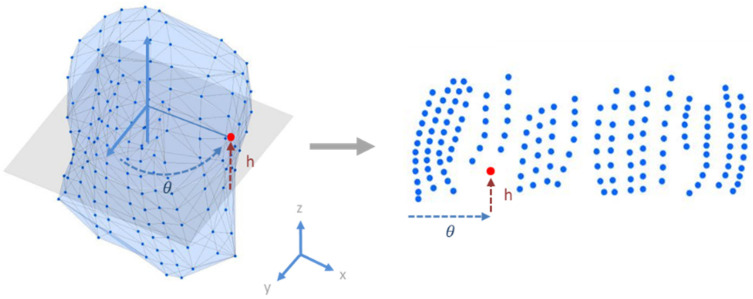
Registration of the torso nodes to the 2D plane. The torso node geometry data were first centered on the geometric origin, and then the nodes were projected to a cylinder surface. 𝜃 is the degree from the *y*-axis to the node and *h* is the height from the *x*–*y* plane to the node.

**Figure 4:**
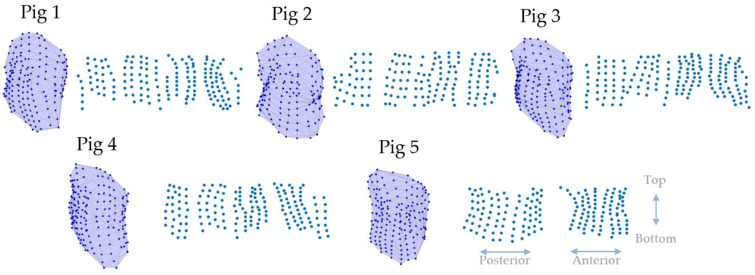
Results of torso node registration to the 2D plane. The 3D view shows the original 3D distribution of the torso nodes, where the ECGs were recorded. The 2D scatter plot shows the distribution of the nodes after projection to a cylinder. The double arrow indicates regions that belong to the anterior, posterior, top, or bottom areas of the torso.

The locations of the torso leads were projected onto a cylinder surrounding the torso. The surface of this cylinder was then used as a sampling plane to produce a 2D image, as shown in Figure 3. The torso node geometry data were first centered on the geometric origin and then the nodes were projected to a cylinder surface surrounding the torso. The nodes’ distance *h* to the *x*–*y* plane then became the vertical distance. The degree θ between the *y*-axis and the node was then the horizontal distance. Figure 4 shows the results of this transformation.

#### 2.4.2. Transforming 1D Data into 2D Data

**Figure 5:**
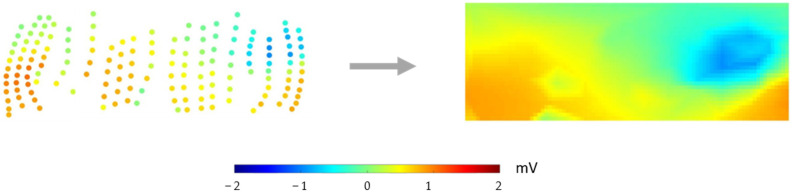
Bilinear interpolation was used to transform the data into 2D format. Left: Normalized torso node distribution. The node color indicates the potential value. Right: Grids with 30 × 90 pixels were merged with the scattered nodes. Nodes in the grid were used as sampling points. The potential values were computed by the bilinear interpolation method. The potential values shown here are data from Pig 1’s first recording at 90 ms after pacing.

Bilinear interpolation was used as the sampling method. The sampling points were depicted on a grid with a width of 90 pixels and a height of 30 pixels, as shown in Figure 5.

#### 2.4.3. Epicardial Surface Node Registration

For the epicardial nodes, the original data were a sequence of electrocardiograms. Each pig had its own sequence, and each electrocardiogram was from a different recording location; there was no geometrical correlation of the sequence of leads.

To unify the data across all pigs’ data, we invented a method of translating the electrocardiograms to the same sequence and number. The overall concept is that we projected the epicardial nodes to the *x*–*y* plane and used a template of node distribution as the sampling point across the projected nodes. Since the sequence of nodes in the template was fixed, we could transform the original data into 1D data in which the data sequence had a fixed geometrical order.

**Figure 6:**
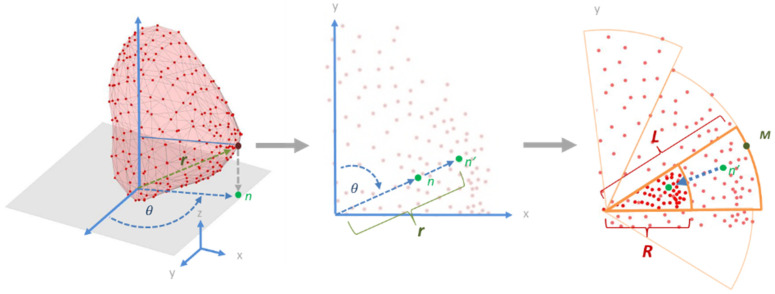
Registration of epicardial notes to the 2D plane. The apex of the heart was used as the origin of the coordinate system. The nodes were then projected onto the *x*–*y* plane at *n* and then the selected node was extended along the vector between the tip and *n* until the distance between the origin and node was *r*, where *r* is the distance between the origin and the epicardial node; the final location is *n’* on the *x*–*y* plane. The scatter plot was further shrunk to a circular area by dividing the map into segments. In each section, we found the node *M* with the maximal radius L and then shrunk this node concentrically to a position with radius *R*. For the rest of the nodes in this segment, we reduced their radius so that the ratio of the new radius to the old radius was retained (equal to *L/R*). The arc of the segment in this figure is 30°.

As shown in Figure 6, the node at the tip of the heart over the apex was used as the origin in the coordinate system. The epicardial node was first projected to the *x*–*y* plane. The result was a scattered map containing the orientation of the node to the body. However, some nodes overlapped with each other, since they could be on top of each other. To have a more evenly distributed map, we extended the nodes’ location along the vector from the origin to the nodes. The result was a 2D scatter map, where the location indicated the orientation of nodes and the distance to the tip of the nodes in the original 3D space.

**Figure 7:**
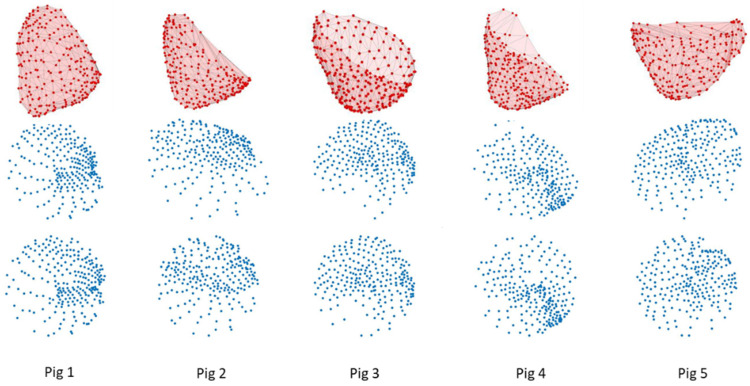
Results of epicardial node registration to the 2D plane. (**Top**): Original 3D distribution of the epicardial nodes. (**Middle**): Initial registration of the nodes. (**Bottom**): Further transformation of the nodes into a circular area.

Since different hearts have different shapes, the distributions of the nodes were not circular but oval. To further normalize the distribution, we further chopped the map into segments. The radius of each segment was the radius of the node that was the most distant from the center *L*. The nodes inside the segment were further shrunk at same ratio toward the center so that all nodes were within a smaller segment with the radius *R*. After this process, all nodes were within a circular area with the radius *R*. When the arc of the segment was set to 30°, this meant that the whole scatter plot was divided into 12 segments; Figure 6 shows this process. Figure 7 shows the epicardial registration results from four pigs.

#### 2.4.4. Transforming 2D Data into 1D Data with the Same Geometrical Sequence

**Figure 8:**
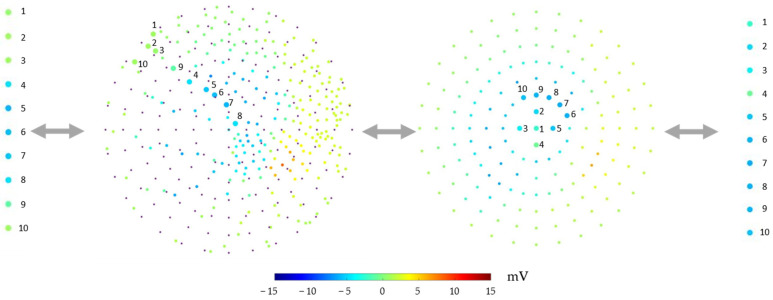
Transformation of 1D data by resampling with the new registration. Far left: First 10 nodes of the original potential data sequence 90 ms after the start of pacing. Middle left: Scatter plot of all epicardial nodes after the new registration. The larger nodes are 10 examples. The small purple nodes are the sampling locations of the template. Middle right: Results from sampling at the locations on the template. The bigger nodes are the first 10 nodes of the template. Far right: The first 10 nodes of the potential data after transformation. The potential values shown here are data from Pig 1’s first recording at 90 ms after the start of pacing.

Each pig had its own sequence during the experiment. After epicardial node registration, we mapped the potential to the 2D scatter plot. We established a template with 165 nodes. The choice of location was rather arbitrary; we used seven layers of circles to cover the region of the 2D scatter plot. These 165 nodes were then used as sampling points to obtain the potential values. We used bilinear interpolation to acquire the data, which were then further transformed into 1D data on the basis of the template’s node sequence. Here, the sequence we used was to put the first node at the center and then gradually move outward in a clockwise pattern. Since the sequence was fixed, even different pigs with different node sequences had similar geometric sequences, as shown in Figure 8.

The overall data flow of Part II was as follows:

1. Transforming the 1D data of the epicardial potential into a 2D scatter plot;
2. Using a template with 165 nodes to sample the potential;
3. Training the model, with the output in the form of 165 1D sequences;
4. During testing, the output of the model was transformed back into the original sequence of epicardial potential by sampling the potential using the 2D scatter plot locations.

#### 2.4.5. Model Selection

**Figure 9:**
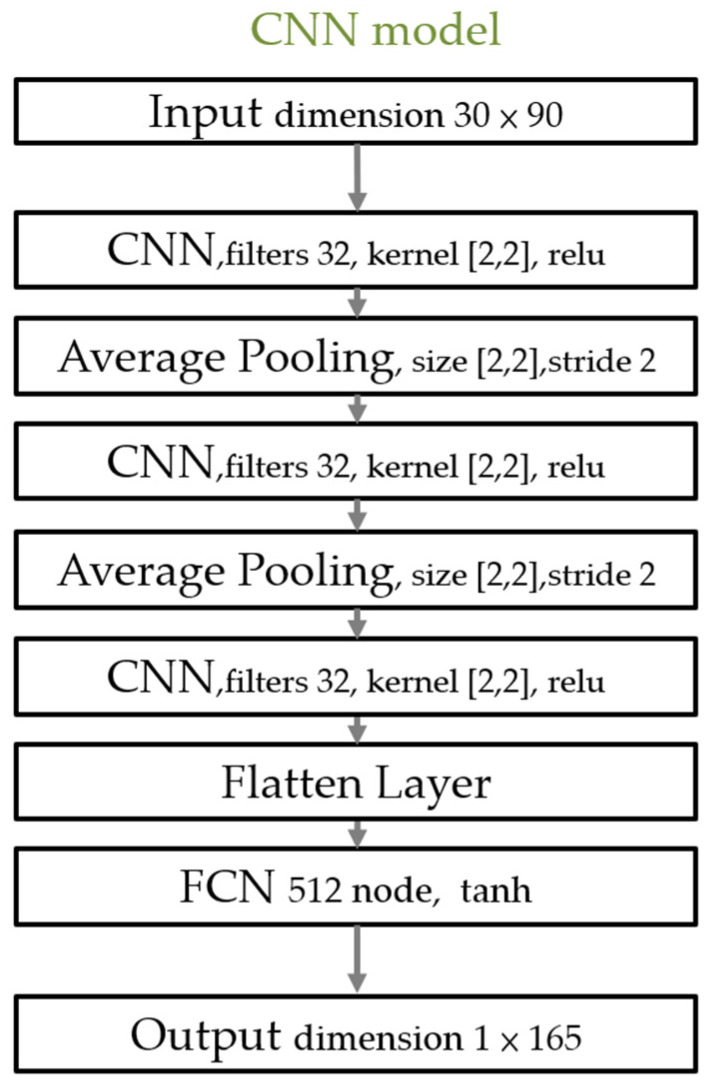
The model used in cross-validation for different pigs. Filter 32 indicates that the output depth of the CNN layer was 32. Tanh is the hyperbolic tangent that was used as an activation function.

We used a simple neural network composed of three layers of a Convolutional Neural Network (CNN). The average pooling layer was also used, as shown in Figure 9. Hyperbolic Tangents were used as the activation function.

### 2.5. Model Evaluation

#### 2.5.1. Leave-One-Out Cross-Validation

In Part I, when geometry was not considered, one full electrocardiogram cycle was the most meaningful representation of the heart activity, so we chose each recording as a single observation. Each recording took its turn being used as the validation data while the rest were used as the training data. For example, if there were 13 recordings from one pig, each recording was used in turn as a validation set while the rest were used as the training set, with 13 trained models in total.

In Part II, each pig had its own geometry. To show how the model performed with different geometries, all recordings from each individual pig were used as a single data unit. Each pig’s data were used in turn as the validation data while the rest of the data were used as the training data. For example, there were 61 recordings from four pigs and 13 recordings from Pig 1. For the first cycle of cross-validation, 13 recordings from Pig 1 were used as validation data while the remaining 48 recordings were used for training. There were four trained models after cross-validation.

#### 2.5.2. Evaluation Metric: Potential Prediction

The correlation coefficient (*CC*) was used to evaluate the predictions of the electrocardiograms for individual leads across all time steps. The *CC* at electrode k is defined as follows:

(3) $$CC_{time-k}=\frac{\sum_{i=1}^{t}\left(V_{M}^{i}-\mu_{M}\right)\left(V_{R}^{i}-\mu_{R}\right)}{\sqrt{\sum_{i=1}^{t}{\left(V_{M}^{i}-\mu_{M}\right)}^{2}}\sqrt{\sum_{i=1}^{t}{\left(V_{R}^{i}-\mu_{R}\right)}^{2}}}$$

where $V_{M}^{i}$ and $V_{R}^{i}$ are the potential at electrode k for the measured (*M*) and reconstructed (*R*) data, respectively; t is the length of the samples (the recorded sequences); and $\mu_{M}$ and $\mu_{R}$ are the corresponding mean values across all samples.

Each recording had 239 correlation coefficients, since there were 239 epicardial leads and, therefore, 239 recordings. For representation, we will only show the mean of all correlation coefficients across these 239 nodes.

#### 2.5.3. Activation Time Reconstruction and Pacing Site Localization

Potential reconstruction is only the first step in monitoring heart activity. To show the potential use of ECGi, we tried to reconstruct the activation time and later predict the initial pacing site.

The activation time (*AT*) is often used to determine the source of pacing. The definition of *AT* at electrode k is as follows:

(4) $$AT_{k}=\underset{t}{\mathrm{argmax}}\left(-\frac{V_{t+dt}-V_{t}}{dt}\right)$$

where $V_{t+dt}-V_{t}$ is the potential difference after one time step for the *t-*th sample. The activation time was the time step that had the maximal voltage decline in one time step. In our study, dt was set to one sampling time: 0.5 ms. The node that had the smallest activation time was then predicted as the pacing site.

#### 2.5.4. Evaluation Metric: Activation Time

The correlation coefficient (*CC*) was also used to assess the accuracy of the predicted activation time, as follows:

(5) $$CC_{AT}=\frac{\sum_{i=1}^{N}\left(AT_{M}^{i}-\mu_{M}\right)\left(AT_{R}^{i}-\mu_{R}\right)}{\sqrt{\sum_{i=1}^{N}{\left(AT_{M}^{i}-\mu_{M}\right)}^{2}}\sqrt{\sum_{i=1}^{N}{\left(AT_{R}^{i}-\mu_{R}\right)}^{2}}}$$

where $AT_{M}^{i}$ and $AT_{R}^{i}$ are the activation time at electrode i for the measured (*M*) and reconstructed (*R*) values, respectively, N is the number of leads, and $\mu_{M}$ and $\mu_{R}$ are the corresponding mean values across all activation times. The activation times were further smoothed by incorporating the global activation fields [16].

#### 2.5.5. Evaluation Metric: Localization Error

Activation times were used to find which node activated first. The node with the smallest activation time was chosen as the initial activating node. The localization error was the Euclidean distance between the node identified by the recorded potential and the node identified by the reconstructed potential. We did not use the real pacing site to calculate the localization error. One reason for this is that we did not have the exact pacing site’s location in the endocardial pacing data. For another, the pacing site predicted by the recorded electrogram was not the same as the real pacing site. To simplify the analysis, we used the pacing site derived from the recorded electrogram as the ground truth.

## 3. Results

### 3.1. Potential Visualization

**Figure 10:**
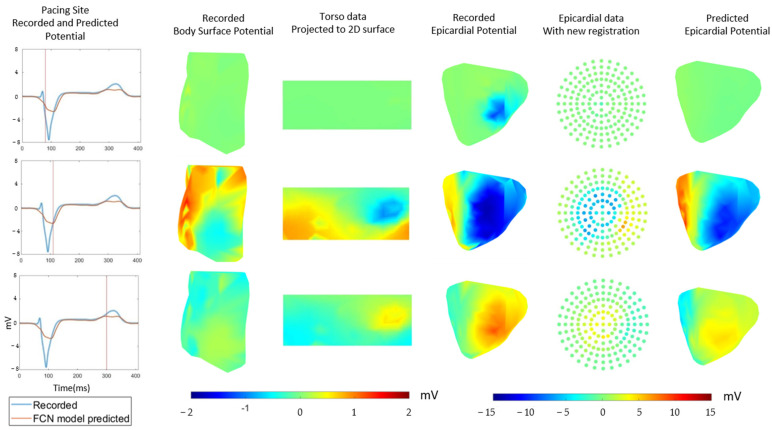
Example of visualizing the potential. The figure shows the potential in three time steps from top to bottom. First column: Electrogram of the recorded and predicted potential. The vertical red line indicates the time step. Second column: Visualization of the torso’s potential. Third column: Potential from the torso lead’s recording after 2D transformation. Fourth column: Visualization of the epicardial potential. Fifth column: Epicardial node potential after transformation into a template. Sixth column: Visualization of the reconstructed epicardial potential.

Figure 10 shows an example of a visualization of the potential. From the top row to the bottom row, the figure shows the potential in three time steps. The top row is during the initial depolarization process, the middle row is during the late depolarization process, and the bottom row is the repolarization phase.

**Figure 11:**
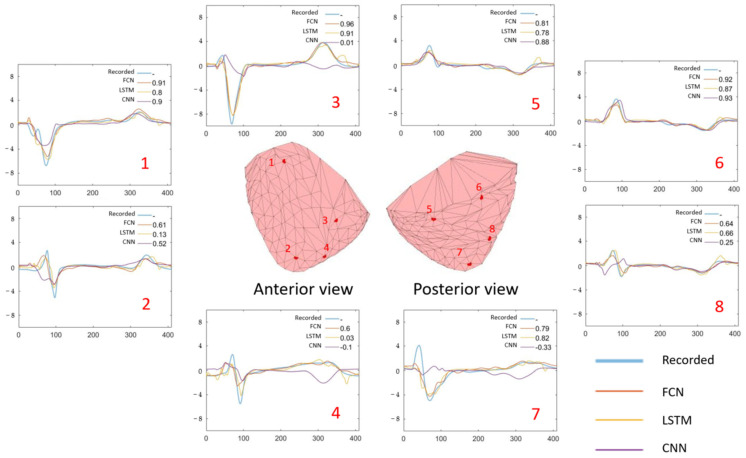
Examples of epicardial site electrograms (recorded and predicted). The corresponding correlation coefficients are also shown in the right upper part of the sub-graphs. The testing data shown here are the first recording from Pig 2. Graph 1–8 shows the recorded and the predicted electro-cardiac waves over 8 epicardial site as examples. The number listed over the right upper corner shows the correlation coefficient between the recorded and the predicted wave from different models. FCN: predicted result from the Fully Connected Neural network for cross-validation within the same pig; LSTM: predicted result from the Long Short-term Memory model for same-pig cross-validation; CNN: predicted result from the Convolutional Neural Network for cross-validation with different pigs.

In Figure 11, the potential from the torso lead recording after 2D transformation is shown in the third column. The fourth column shows the epicardial node’s potential after its transformation into a template. The rightmost column shows the reconstructed potential.

### 3.2. Median Correlation Coefficient

**Figure 12:**
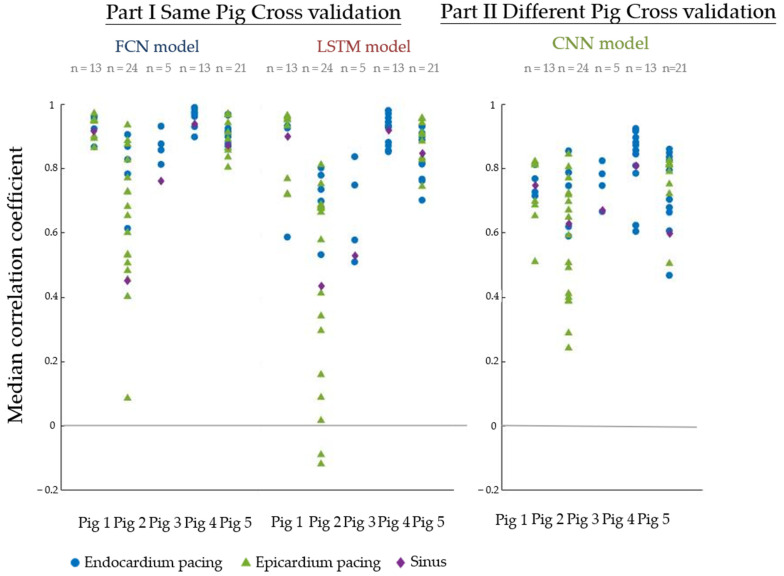
Cross-validation results. (**Left**): Cross-validation results using data from the same pig. (**Right**): Cross-validation results using data from all pigs. Each dot represents the median correlation coefficient across 239 epicardial nodes.

Figure 12 shows the cross-validation results. For example, the dot in the figure for Part I for the FCN model testing results represents one of the cross-validation model test results. The dot represents the median correlation coefficient across all epicardial nodes $CC_{time-k}$ as indicated in Formula (1). The model trained from Pigs 2–4 was tested on the data from Pig 1. Each recording had one median correlation coefficient, so there are 13 dots shown in Part II for the CNN model’s first strip of dots.

The results show that when we considered data from only a single pig, the performance was quite varied. For example, the Fully Connected Neural network (FCN) and the Long Short-term Memory network (LSTM) performed well in Pigs 1 and 3 with median correlation coefficients of >0.8. If we accumulated all the correlation coefficients from all results, the overall medians of the correlation coefficient and the first to third quantiles were 0.90 [0.68–0.96] and 0.82 [0.54–0.93] for the FCN and LSTM, respectively.

When all data were combined, the overall performance was poorer. If we accumulated all the correlation coefficients from all results, the overall median of the correlation coefficient and its first to third quantiles was 0.74 [0.22–0.89]. This is shown in the right-hand part of Figure 11.

### 3.3. Activation Time Correlation

**Figure 13:**
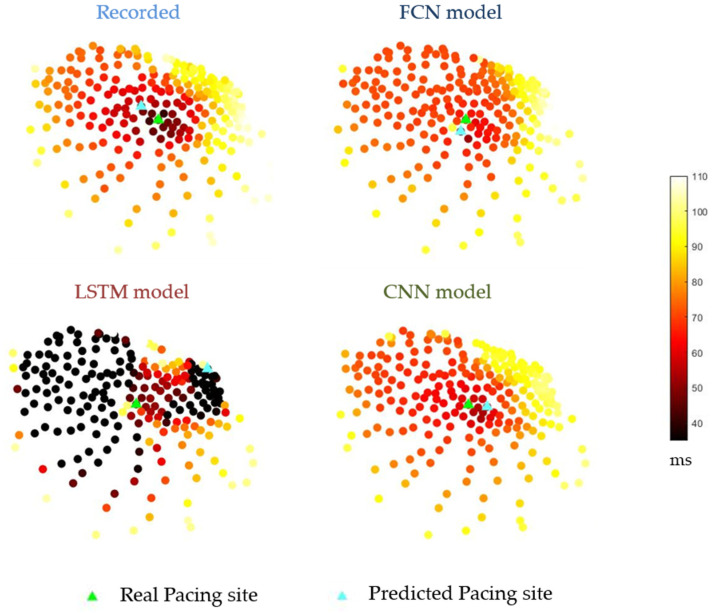
Examples of activation time maps, shown as scatter plots. The recording here is from Pig 2. Recorded: The activation map derived from the recorded electrogram. The spots represent one node on the epicardial surface. The colors indicate the value of the activation time. FCN model: The activation map derived from the electrocardiogram reconstructed by the Fully Connected Neural network model. LSTM model: The activation map derived from the electrogram reconstructed by the Long Short-term Model. CNN model: The activation map derived from the electrogram reconstructed by the Convolutional Neural Network model. Green triangles: real pacing site in the experiment; light blue triangles: predicted pacing site with the lowest activation time.

Figure 13 is an example of an activation time map. The darker areas indicate a shorter activation time. In the figure, note that even the activation time derived from the recorded electrocardiogram could find the real pacing site.

**Figure 14:**
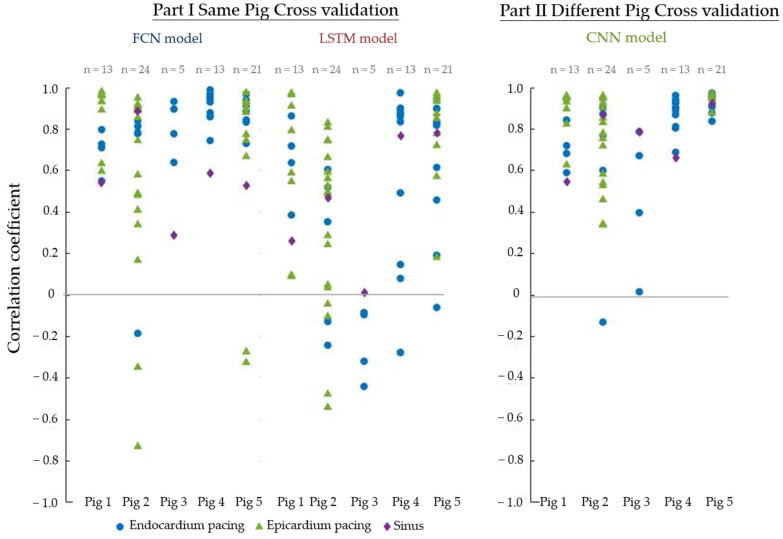
Correlation coefficients between the activation time maps derived from the recorded data and the reconstructed electrogram. (**Left**): Correlation coefficients of the activation time map for the validation data after cross-validation. (**Right**): Correlation coefficients of the activation time maps of all validation data in the cross-validation. Each dot represents a correlation coefficient between the activation time map from the recorded data and the reconstructed data.

The activation time map derived from the recorded data was compared with that from the reconstructed data, as shown in Figure 14. The results from Part I showed a wide variation for different data: while some of the data showed a correlation coefficient of up to 0.9, many of the data showed negative correlations. The overall medians of all correlation coefficients and the first to third quantiles are 0.86 [0.61–0.93] and 0.52 [0.05–0.80] for the FCN and LSTM models, respectively. Part II, which combined all the available data, showed a much better performance, with only one result below 0. The median of all correlation coefficients, along with its first to third quantiles, was 0.82 [0.67–0.93].

### 3.4. Localization Error

**Figure 15:**
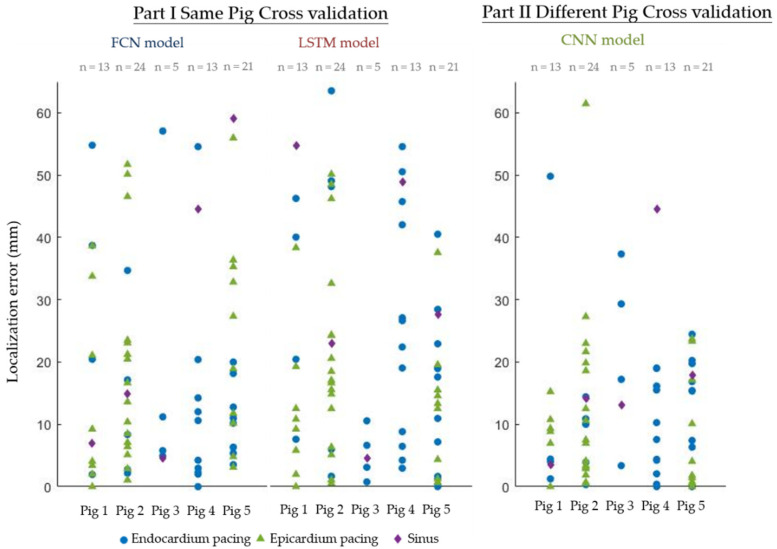
Localization error. (**Left**): Localization error of cross-validation of the data from the same pig. (**Right**): Localization error of cross-validation with different pigs.

Figure 15 shows the localization error of different cross-validation results. The results from Part I showed great variation for different data: some located the exact same node as the initial activation but some located an area around 60 mm distant. The medians of all localization errors and the first and third quantiles are 10.4 [3.6–22.6] mm and 18.5 [6.4–41.5] mm for the FCN and LSTM models, respectively.

The performance was much better for the results from Part II. All the localization errors and the first and third quantiles of all localization errors were 9.3 [3.4–17.0] mm.

## 4. Discussion

### 4.1. Interpretation of the Results

One of the main advancements in this study is that it solved the overfitting problem. Currently, neural networks have sufficient flexibility to fit nearly all data types. However, a common problem is that the model cannot generalize to data that the model has not seen. During training, we observed that even when using a simple neural network with one fully connected layer, the model converged to obtain a correlation coefficient of nearly 1. However, the overfitting problem always occurred.

In our dataset, the pattern of pacing had sufficient variability in the pacing site across all areas of the heart’s surface and endocardium. This probably explains why our results were much better than those of previous studies using neural networks. This may also explain the better results in Part II, which incorporated much more data with different pacing sites.

When viewing the results, we can see that the variability between different pigs and different recordings varied greatly. For example, in Part II, the CNN model performed very well when cross-validated on Pig 5, showing correlation coefficients all above 0.8. However, in Pig 2, some recordings even showed negative correlations. This may be due to the small dataset. The dataset was not large enough to cover all the possible variability in a heart condition. Another observation is that if we compare the results from Part I and Part II, the differences can be explained by the fact that in Part II, we incorporated data from different pigs, which greatly increased the amount of training data.

### 4.2. Previous Studies Using Machine Learning and Deep Learning for Inverse Problem

In recent years, there has been a growth of interest on using deep learning techniques for inverse problem. Currently, most of the study focused on image reconstruction for magnetic resonance imaging (MRI) or computed tomography (CT) [17]. The task for using these techniques include denoising, deconvolution, supper-resolution and medical image reconstruction. This trend has also expanded into the field for other physics model reconstructions [18,19,20]. They use machine learning and a deep learning range from finding the operator (as the A described in formula (1) in Section 1.3) to regularize the output, or they could be used for directly pairing a target and measurements when the model is unknown or difficult to obtain [21].

Probably due to scarcity of the data and difficulty in modeling, currently there are only very few studies focusing on using deep learning for the inverse problem of heart surface potential reconstruction. In our study, we present a method to solve the heart surface potential inverse problem by building a model by directly pairing ground truth and measurement. We also proposed a method to align data from different geometries by transforming 1D data into 2D data, which also would allow the deployment of CNN.

### 4.3. Comparison with Previous Reported Accuracy

Most previous studies have median correlation coefficients of around 0.7. With a relatively simple model, we achieved an overall correlation coefficient of around 0.74, see Table 2. There were only five pigs, which means that the geometrical variance may not be sufficient to obtain a better prediction. Considering that our dataset was relatively small, this result is quite promising.

### 4.4. How Important Is the Geometrical Information?

We showed that a rough geometrical transformation was sufficient for a model to make predictions. In the transformation of torso node information, the projection of nodes to the cylinder’s surface will definitely eliminate some information. For the transformation of the heart nodes, the distortion was even greater, and the registration did not consider the position of the heart in the body. However, the results showed that the information was sufficient to provide a model that can make predictions and avoid overfitting.

### 4.5. Potential Clinical Application

The dataset we used in this study was epicardial pacing. The corresponding disease condition was idiopathic ventricular tachycardia, which is an abnormal excitation of the ventricle without a structural problem. Most of the time, idiopathic ventricular tachycardia presents with a single premature ventricular contraction in body surface ECGs. This propagation of potential over heart is the same as the propagation elicited from an electrode pacing in our study. Sometimes, it shifts to the consecutive form and shows as ventricular tachycardia in body surface ECGs. The treatment is ablation of the abnormal excitation site, so localization of this abnormal pacing site is crucial for performing this surgery. Usually, the process of localization is carried out through electroanatomical mapping before ablation. However, if the localization can be achieved non-invasively, it can be used for pre-surgical evaluations to narrow down the area for pacing site mapping. Furthermore, it can even be combined with radiation therapy to eliminate the need for surgery altogether [28].

This study shows the potential to identify the origin of premature ventricular activation through non-invasive electrocardiogram recordings of the body’s surface. The information can be used to assist the identification of ablation sites.

This result shows the possibility of avoiding the need for geometric information. For example, we could obtain a standard torso and heart as a template for registration of an electrode’s 3D location. When a new patient is being tested, the electrode’s position will then be registered on the standard body and the predicted epicardial potential can be viewed on the standard heart. With this workflow, we can avoid the need to perform CT or MRI examinations. However, this will need a lot more study to be realized.

### 4.6. Limitations

Our dataset still has a limited scope. It did not contain rhythms such as atrial fibrillation, ventricular fibrillation, or ventricular premature beats. In addition, it did not contain data from hearts with scarring. Therefore, the model can only be applied to conditions such as sinus rhythm or epicardial pacing. Furthermore, due to the scarcity of data containing simultaneous recordings of body surface and heart potentials, we could not validate our method with another independent dataset.

## 5. Conclusions

A neural network can be used to solve the inverse problem of ECGi with relatively small datasets. Our best result showed the overall median of the correlation coefficient to be 0.82. Our study also showed that rough geometrical information of the torso and heart may be enough to reconstruct the epicardiogram. The performance of the model was inconsistent between different recordings and pigs. This may be due to the relatively small dataset and may improve with a larger dataset. As shown in Part II, a better result was obtained when the model was trained with more data. In a clinical setting, this study shows the potential to identify the source of a pacing site through a non-invasive electro-cardiogram recorded at the surface, which can be applied for evaluation of a patient with premature ventricular contractions.

## Figures and Tables

**Table 1:** Number of recordings from different pacing sites and recording leads. The first three rows showsthe number of recordings with different pacing sites or rhythms. The bottom two rows show the number of recording leads placed over the torso and epicardial surfaces.

	Pig 1	Pig 2	Pig 3	Pig 4	Pig 5
Sinus rhythm	1	1	1	1	1
Endocardial pacing	4	5	0	12	10
Epicardial pacing	8	18	4	0	10
Total number	13	24	5	13	11
Torso lead number	158	150	171	165	170
Epicardial lead number	239	239	239	239	239

**Table 2:** Comparison with previous studies for the reconstruction of epicardial potential. This table is adapted from Table 2 with permission from Bear et al. [11]. The results are presented as the mean ± SD or the median [interquartile range]. * Only the results from paced data are show. ** The results shown here are from the CNN model in this study.

Subject Type	Subjects	ECG Cycles	Electrogram Correlation Coefficient	Localization Error mm	Activation Time Correlation	Reference
Torso tank		4	>0.8	2–10		[1,22]
Human	3	5	0.72 ± 0.25	13 ± 8		[23]
Human	4	79		13 ± 9		[24]
Human *		6			0.68 ± 0.17	[12]
Human	4	46		20.7 [9.6–33.2]	0.71 [0.65–0.74]	[25]
Dog	4	93	0.71 [0.36–0.86]	10 [7–17]	0.82	[26]
Pig	9	118		20.7 [13.8–25.6]		[27]
Pig	5	70	0.72 [0.40–0.84]	16 [9–26]	0.78	[11]
Pig **	5	71	0.74 [0.22–0.89]	9.3 [3.4–17.0]	0.82 [0.67–0.93]	This study

## Data Availability

A subset of the experimental data acquired in this study has been made available for the electrocardiographic imaging community through the EDGAR project (Experimental Data and Geometric Analysis Repository; http://www.ecg-imaging.org/ (accessed on 25 February 2022).
